# Relieving the pediatric emergency department by referring low triaged patients using the Manchester Triage System

**DOI:** 10.1007/s00431-025-06230-5

**Published:** 2025-06-10

**Authors:** Anna Sorz-Nechay, Franziska Leeb, Michaela Haas, Emina Djordjevic, Lusine Yeghiazaryan, Susanne Greber-Platzer

**Affiliations:** 1 Division of Pediatric Pulmonology, Allergology and Endocrinology, Department of Pediatrics and Adolescent Medicine, Medical University of Vienna, Waehringer Guertel 18-20, 1090 Vienna, Austria; 2 Center for Medical Data Science, Institute of Medical Statistics, Medical University of Vienna, Spitalgasse 23, 1090 Vienna, Austria

**Keywords:** Pediatric emergency medicine, Manchester Triage System, Patient safety, Patient referral, External medical center

## Abstract

In 2015, the pediatric emergency department (PED) of the Department of Pediatrics and Adolescent Medicine, Vienna General Hospital, reached its capacity with 25,000 patient visits, leading to delays in assessment and overcrowding. Self-referred patients constituted 95%, with 75% classified as low urgency level 4 (UL-4; green) and 5 (UL-5; blue) according to the Manchester Triage System (MTS). In 2016, a referral process was established in collaboration with local pediatric offices and the pediatric departments of the Vienna Hospital Association to refer low-triaged patients for medical assessment, reducing total visits to 16500 by 2017. This study aimed to evaluate patient safety, satisfaction, and diagnosis of referred patients. Between June 2017 and May 2018, UL-4 and UL-5 referred patients were included (*n* = 2394). Data on diagnosis, treatment, and satisfaction were collected via a survey from 568 patients (23.7%). Of all referred patients, 6 (1.1%) required hospitalization, with no significant differences in hospitalization rates across age groups (*p* = 0.861) or gender (*p* = 0.766). The most common medical issues were respiratory diseases, both in outpatients (*n* = 198/562, 35.6%) and inpatients (*n* = 4/6, 66.7%). Families’ satisfaction levels with the process at the PED and external treatment were high (*n* = 530, 93.3% and *n* = 477, 84.0%, respectively). Dissatisfaction was higher with the PED (*n* = 26, 4.6% vs. *n* = 23, 4.0%), linked to unclear referral communication. *Conclusion*: The referral of low-urgency patients using the MTS can reduce PED congestion, without compromising patient safety and satisfaction levels.

**Table:** 

What is Known:
• The MTS is widely used as a valid and reliable tool in PEDs.
• Overcrowding in PEDs is a common issue, leading to the need for alternative management strategies, particularly for nonurgent cases.
What is New:
• The burden on the overcrowded PED can be effectively alleviated by redirecting low-triaged pediatric patients (UL-4 and UL-5) using the MTS to primary or secondary care medical centers.
• The study provides evidence that referred pediatric patients and their families experience satisfaction with provided care and that there is no relevant increase in hospitalization rates or adverse outcomes.

## Introduction

Triage systems are essential in emergency departments (ED) to ensure safe and efficient management of patient flow based on clinical priority [1–3]. The Manchester Triage System (MTS) utilizes a structured approach, incorporating general and symptom-based flowcharts, including some specific ones for pediatric patients, and discriminators to categorize patients into five urgency levels (UL) [4]. Each UL is numbered and color-coded and corresponds to a maximum time frame within which a patient must be reassessed or referred to the physician: UL-1 (red, emergent, 0 min), UL-2 (orange, very urgent, 10 min), UL-3 (yellow, urgent, 60 min, 30 min in the German translated edition), UL-4 (green, less urgent, 120 min original version, 90 min in the German translated edition), and UL-5 (blue, non-urgent, 240 min, 120 min in the German translated edition) [4]. Patients categorized under UL-1 (red) require immediate treatment [4, 5]. The validity of the MTS in pediatric EDs (PEDs) is considered to be moderate with a tendency towards over-triage [6–9].

The Emergency Unit of the Department of Pediatrics and Adolescent Medicine at the Medical University of Vienna primarily treats self-referred patients up to the age of 18 years. As a tertiary care center, about one-quarter of the patients presenting to our PED have chronic conditions.

In 2015, due to a continuous increase in patient numbers with approximately 25,000 annual presentations, our PED reached its staff capacity limits. In the first half of 2016, the outpatient clinic had to manage up to one hundred patients daily during peak times. Consequently, we observed triage delays after administration, prolonged waiting times, overcrowding in the waiting areas, limited availability of examination rooms, and staff exhaustion. During times of reduced medical coverage outside regular working hours and on weekends, physicians reached their working limits and had no option to order additional medical support. The surge compromised the ability to adequately care for patients with chronic illnesses, necessitating the exploration of strategies to alleviate the burden on the PED.

In our center, self-referred patients accounted for 95% of the PED visits, with approximately 75% classified as UL-4 and UL-5 according to the MTS. To address this, a referral system for low-acuity pediatric patients was implemented in September 2016 in collaboration with public hospitals of the Vienna Hospital Association (German: *Wiener Gesundheitsverbund*) and pediatric doctor’s offices. This represents a graded medical care system with cooperation between the university hospital as a tertiary care center and pediatric doctor’s offices and public hospitals as the primary and secondary medical care centers, respectively. The referral of non-urgent cases to external medical centers could improve the critical situation in the PED and guarantee immediate medical care for severe and critically ill pediatric patients in the tertiary care center. As an additional positive aspect, we observed a significant reduction of self-referred patients within a few months, especially among low triaged children and adolescents, with the total number of visits decreasing to 16,500 in 2017.

In Austria, EDs are established in all public hospitals and medical care is offered 24 h a day, every day of the year, for patients with acute illnesses, general health problems, including any discomfort, and emergency cases. General practitioners and pediatric doctors’ offices should be the first address contacted in case of any illness, except life-threatening events or critical illnesses. Nevertheless, many patients prefer to seek care directly in emergency departments, mainly due to unrestricted access, round-the-clock availability, and the possibility of having all examinations on site. Moreover, healthcare insurance is compulsory for all residents and is regulated by Austrian law. This allows patient visits in EDs via self-referral, and the public hospitals must offer medical care to all. While the system seems to be patient-oriented and safe, it contributes to overcrowding, especially in tertiary care centers.

Previous studies have explored the effectiveness of direct admission of low-triaged patients to doctors’ offices, mainly in the adult population, and initiated by prehospital emergency services or general practitioners in EDs [10–13]. However, data are missing in graded medical healthcare for MTS-based referral of low-triaged pediatric patients from a tertiary care center to pediatric doctors’ offices and public hospitals, representing primary and secondary medical care, respectively. To our knowledge, no previous studies have assessed examinations, diagnoses, and outcomes of referred pediatric patients, nor parents’/caregivers’ satisfaction with the referral process, focusing on the main goal of risk stratification for pediatric patients and acceptance among parents/caregivers.

## Material and methods

### Study design and study population

This retrospective analysis included 2394 patients who visited the PED of the Department of Pediatrics and Adolescent Medicine at the Medical University Vienna and were further referred to external medical centers between June 2017 and May 2018. Referral of patients was considered for those triaged as low urgency, specifically categorized as UL-4 and UL-5 according to the MTS, and was coordinated in collaboration with local pediatricians and the pediatric departments of the Vienna Hospital Association. Hospital admission as a result of the presented illness at the triage within the next 14 days served as a reference standard for identifying more severe outcomes in referred pediatric patients.

Before implementing the referral process, in-house clinical psychologists were consulted to develop patient- and parent-friendly formulations for the triage nurses for effectively communicating the PED physicians’ decision regarding referral. Working as a triage nurse was only possible for staff nurses with a minimum of three years’ experience in the PED and MTS basic course certification.

The MTS was utilized for risk stratification, and the decision for external referral was made by PED physicians based on the electronic triage documentation, without direct patient contact. Newborns, patients with certain chronic illnesses [14], those managed in our specialized outpatient clinics, patients with suspected highly infectious diseases, patients waiting longer than 15 min after the initial MTS assessment for a physician’s referral decision, and patients/families with major language barriers were excluded from the referral.

Following the triage assessment, the PED physician reviewed the MTS documentation for low-acuity patients and, if confident that a referral could be made safely, activated the referral process by clicking the designated button in the electronic patient managing system. Afterwards, PED triage nurses provided parents/caregivers with an informed consent to sign, confirming that they were informed about the referral system, along with the triage documentation and a list of external medical centers. In general, referrals were made to pediatric doctors’ offices during regular working hours and to the PEDs of public hospitals outside regular working hours and on weekends. Referred patients were advised to proceed to the external medical center without delay, by public or private transport. Data on parental refusal to sign the informed consent and disclaiming the referral were not systematically recorded. In such rare cases, the triage nurse notified the PED physician, who had to explain the referral process and the decision to the parents/caregivers. In most cases, consent was subsequently obtained. Otherwise, the PED physician had to decide whether to perform a brief assessment, initiate treatment, or wait for a second triage with reassessment for potential referral.

Some months later, all referred patients/caregivers were informed about the study evaluating the external referral process through a letter that included the study description, an invitation to participate in the survey, the survey itself, and patient/caregiver informed consent forms.

Subsequently, a telephone follow-up survey was conducted to gather information on diagnosis, outcomes, and satisfaction with provided information and treatment. The questionnaire was available in German, English, Turkish, and Arabic. The telephone calls were conducted by three medical students, all fluent in German, and additionally in English, Turkish, or Arabic. The medical students were trained in appropriate communication techniques by in-house pediatric clinical psychologists. Each call followed a standardized protocol, providing information about the study and inviting participation in surveys on diagnosis, outcome, and satisfaction, which were directly completed with one caregiver.

### MTS and current version

Since 2012, the Pediatric Emergency Unit of the Department of Pediatrics and Adolescent Medicine at the Medical University Vienna has been using the MTS and has utilized the third German edition of the international Manchester triage book published as *Emergency Triage* by the Manchester Triage Group (3rd edition) during the study period 2017–2018 [15, 16]. In 2021, the PED transitioned to using the current fourth edition [4].

### Statistical analysis

Descriptive statistics were used to summarize characteristics of patients included in the study and those lost to follow-up, and variables were compared between groups using *χ*^2^ tests or Fisher’s exact test. Descriptive analyses were conducted with proportions and absolute frequencies for diagnosis, outcome, and satisfaction. Donut charts, bar charts, and tables were used for visualization of the data. The patients were additionally categorized into five age groups (28 days to < 3 months, 3 months to < 1 year, 1–4 years, 5–11 years, 12 years to < 18 years). A *χ*^2^ test was performed to compare the observed hospitalization rates and drug treatment at home across all groups. Additionally, a pairwise post-hoc examination was performed to assess which specific age groups were significantly different, with Bonferroni correction for multiple testing. Statistical significance was determined at a *p*-value of < 0.05. The length of hospital stay was summarized using medians and minimum–maximum values. Statistical analyses and data plotting were performed using Python (version 3.11.7), and the packages scipy [17] (version 1.11.4), Plotly (version 5.9.0), and Matplotlib (version 3.8.0) were utilized.

### Data collection

Triage data were electronically imported, while survey data were manually entered in Microsoft Excel. Access to the data collection was restricted to authorized staff only. The study was approved by the Ethics Committee of the Medical University Vienna, Austria (No. 1151/2018).

## Results

### Patient characteristics

In total, 16,825 patients visited the PED between June 2017 and May 2018. Of those, 10,901 patients (64.8%) were excluded from referral due to the MTS UL-1–3, and 3530 patients (21.0%) met other exclusion criteria. Consequently, 2394 patients (14.2%) were referred to an external medical center. Of these, study data were missing for 1826 referred patients (76.3%) due to the unavailability of the caregivers to participate in the telephone survey. Caregivers of 568 referred pediatric patients (23.7%) completed the survey, and these cases were included in the final data analysis.

Detailed information on the total referred population is presented in Table 1.

**Table 1:** Characteristics of the total referred population. *VAS*, visual analogue scale

	*N* (%) study population	*N* (%) lost to follow-up
Referred patients	568 (100)	1826 (100)
Gender		
Male	297 (52.3)	976 (53.5)
Female	271 (47.7)	850 (46.5)
Age group		
28 d to < 3 mo	10 (1.8)	45 (2.5)
3 mo to < 1a	44 (7.7)	167 (9.2)
1a to 4a	247 (43.5)	782 (42.8)
5a to 11a	187 (32.9)	528 (28.9)
12a to < 18a	80 (14.1)	304 (16.6)
Primary (chronic) disease		
Yes	63 (11.1)	158 (8.7)
No	505 (88.9)	1668 (91.3)
MTS urgency level		
4	529 (93.1)	1695 (92.9)
5	39 (6.9)	131 (7.1)
Fever (> 38.5°)		
Yes	2 (0.4)	5 (0.3)
No	566 (99.6)	1821 (99.7)
Pain (VAS)		
0	313 (55.1)	1006 (55.1)
1–2	91 (16.0)	304 (16.6)
3–4	158 (27.8)	504 (27.6)
5–6	6 (1.1)	10 (0.5)
7–8	0 (0.0)	2 (0.1)
9–10	0 (0.0)	0 (0.0)

**Fig. 1:**
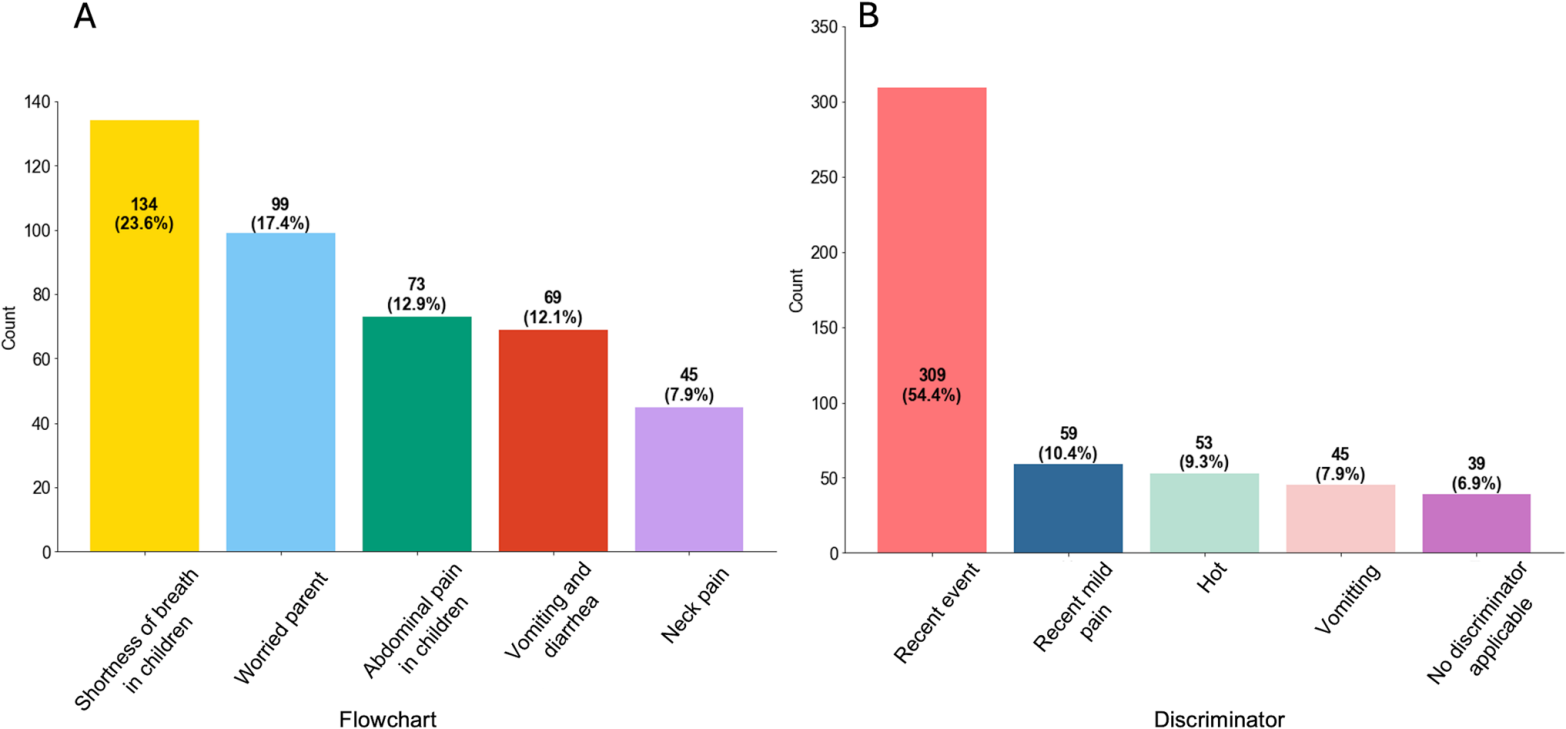
Frequencies and numbers (percentages) of **A** the five most used MTS flowcharts and **B** the five most used MTS discriminators

The majority of referred patients were triaged to UL-4 (*n* = 529, 93.1%). The most commonly used MTS flowcharts were shortness of breath (*n* = 134, 23.6%), worried parent (*n* = 99, 17.4%) and abdominal pain in children (*n* = 73, 12.9%; Fig. 1A). “Recent event,” defined as symptoms and events that occurred within the last 7 days, was the most frequent MTS discriminator (*n* = 309, 54.4%). This was followed by discriminators “recent mild pain” (*n* = 59, 10.4%), characterized by a visual analogue scale (VAS) 1–4, and “hot” (*n* = 53, 9.3%), defined as a fever ranging from ≥ 38.5 to 41.0 °C (Fig. 1B).

### Satisfaction with the referral process at the PED

**Fig. 2:**
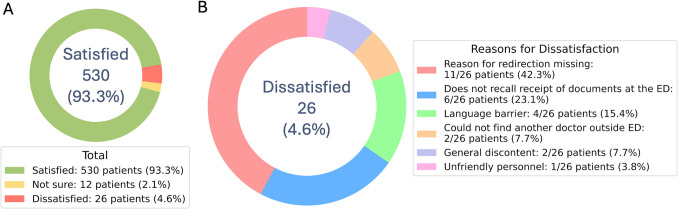
Donut charts, showing frequencies and numbers (percentages) of **A** perception of satisfaction with the referral process at the PED and **B** primary reasons for dissatisfaction with the referral process at the PED

At the PED, parents/caregivers of 530 patients (93.3%) reported satisfaction with the referral process, 12 (2.1%) were undecided, while 26 (4.6%) expressed dissatisfaction (Fig. 2A). Participants who were dissatisfied were asked to specify a single primary reason. The main reason for dissatisfaction was a lack of explanation from the PED triage staff regarding the referral decision of the PED physicians (*n* = 11/26, 42.3%), followed by not recalling receipt of any written documents at discharge from PED, including triage documentation and a list of nearby available medical centers (*n* = 6/26, 23.1%). Language barriers were the third most common cause (*n* = 4/26, 15.4%) and caregivers of two patients (*n* = 2/26, 7.7%) expressed general discontent with the fact that the patients were not seen by an on-site PED physician (Fig. 2B).

### Satisfaction with external care

**Fig. 3:**
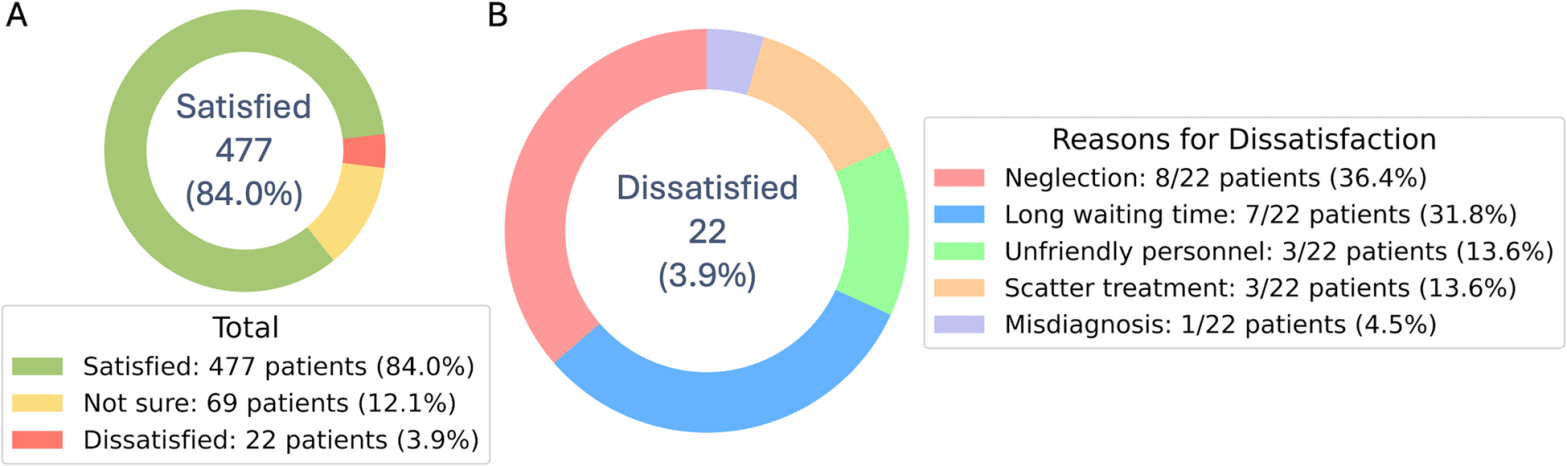
Donut charts, showing frequencies and numbers (percentages) of **A** perception of satisfaction with treatment in the external medical centers and **B** primary reasons for dissatisfaction with treatment in the external medical centers

Overall, caregivers of 477 pediatric patients (84.0%) were satisfied with the external care, 69 (12.1%) were undecided, and 22 (3.9%) were dissatisfied (Fig. 3A). The most common reasons for dissatisfaction were the perception that the patient did not receive adequate care (*n* = 8/22, 36.4%) and long waiting times (*n* = 7/22, 31.8%). Caregivers of three patients (13.6%) expressed dissatisfaction as medical examination and treatment took place at multiple medical centers (Fig. 3B). All caregivers who were dissatisfied had children triaged to UL-4.

### Further examination, treatment, and diagnosis

A total of 478 participants (84.1%) followed the instructions to visit a pediatric doctor’s office or a public hospital in proximity to their home district. Imaging examinations were performed on 24 patients (4.2%). In 384 cases (67.6%), it was reported that the pediatric patient needed to take medication at home. The most prescribed drugs were antipyretics/analgesics (*n* = 72, 12.7%), followed by antibiotics (*n* = 68, 12.0%) and creams or salves (*n* = 25, 4.4%; Table 2). The chi-square test revealed a statistically significant association between five age groups and the decision to give the pediatric patients medications at home (χ2 = 31.05, *p* < 0.001). A pairwise post hoc analysis revealed a statistically significant difference between patients aged 1 year to 4 years and patients aged 12 years to < 18 years (*n* = 190, 83.7% vs. *n* = 41, 62.1%, *p* = 0.002), indicating that older pediatric patients were less likely to receive medication at home. This was further confirmed by performing chi-square analysis between the oldest patient group (12 years to < 18 years) against all younger aged groups merged together (*n* = 341, 79.3%, *p* = 0.003). There was no significant difference between different age groups and antibiotic treatment at home (χ2 = 11.13, *p* < 0.190).

**Table 2:** Frequencies and numbers (percentages of total cohort, *n* = 568) of cases requiring medication at home, including the most prescribed medications, further medical examinations, hospitalization and/or surgery after referral from the PED

	*N*	%
Required medication at home	384	67.6
Antibiotic therapy	68	11.9
Antipyretics/analgesics	72	12.7
Creams/salves	25	4.4
Required further examination	124	21.8
Blood draw	101	17.8
Radiology exams	24	4.2
Hospitalization	6	1.1
Operation	1	0.2

Of the total, six patients (1.1%) required hospital admission within 2 weeks after referral, with all of them classified as UL-4. The mean length of stay was 5 days, ranging from a minimum of 3 days to a maximum of 10 days. None of the patients required ICU admission. Four days after referral from the PED, one patient (0.2%) underwent surgery to drain an abscess that developed on the nose following an insect bite. The detailed information on the six hospitalized patients is provided in Table 3*.* There was no difference in hospitalization rates between the five age groups (χ2 = 1.30, *p* = 0.861) and gender (χ2 = 0.09, *p* = 0.766).

**Table 3:** Detailed information on the six hospitalized pediatric patients

*N*	Age	Gender	Diagnosis	Hospitalization (days)	Operation	MTS flow chart	MTS discriminator	MTS Urgency level	Satisfaction with treatment at discharge from PED
1	9 mo	F	Acute gastroenteritis (A08)	4	No	Vomiting and diarrhea	Vomiting	4	Yes
2	15 y	M	Abscess, furuncle and carbuncle of nose (J34, D21)	3	Yes	Facial problems	Recent event	4	Yes
3	1 y	M	Acute respiratory tract infection (J06)	3	No	Neck pain	Recent event	4	Yes
4	4 y	M	Pneumonia, organism unspecified (J18)	10	No	Shortness of breath in children	Recent event	4	No
5	9 mo	M	Acute bronchitis (J20)	3	No	Shortness of breath in children	Hot	4	Yes
6	10 y	F	Dysphonia, laryngitis, vocal cord dysfunction (R49)	7	No	Neck pain	Recent event	4	Yes

Respiratory tract diseases were the most common condition among referred pediatric patients (*n* = 202/568; 35.6%), being the leading medical issue, applicable to outpatients (*n* = 198/562; 35.2%) or hospitalized patients (*n* = 4/6; 66.7%). This was followed by gastrointestinal diseases (*n* = 69/568; 12.2%) and ear, nose, and throat conditions (*n* = 22/568; 32.2%).

**Fig. 4:**
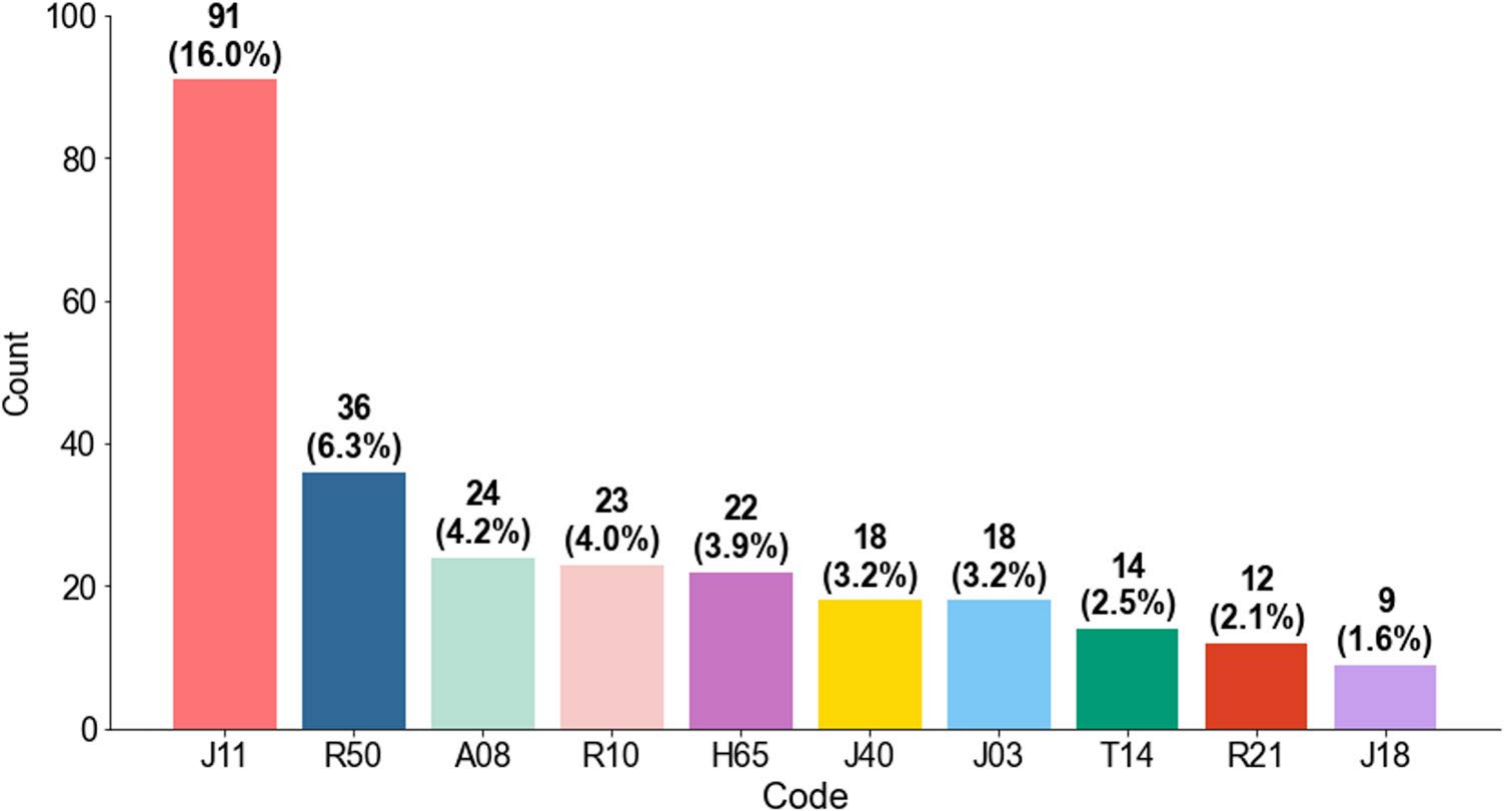
Bar chart showing frequencies and numbers (percentages) of the most common ICD-10 codes (J11, influenza, virus not identified, R50, fever of other and unknown origin; A08, viral and other specified intestinal infections; R10, abdominal and pelvic pain; H65, nonsuppurative otitis media; J03, acute tonsillitis; J40, bronchitis, not specified as acute or chronic; T14, injury of unspecified body region; R21, rash and other nonspecific skin eruption; J18, pneumonia, organism unspecified)

The most frequently used ICD-10 codes were influenza (*n* = 91/568; 16.0%), fever (*n* = 36/568; 6.3%), and intestinal infection (*n* = 24/568; 4.2%; Fig. 4).

## Discussion

This study evaluated the efficacy and safety of the MTS in risk stratification and identification of pediatric patients appropriate for referral from the PED of a tertiary care center to primary and secondary care centers. The referral of 568 patients triaged as UL-4–5 was feasible, as evidenced by clinical stability and a low risk of hospitalization. Within 2 weeks following the referral, six patients (1.1%) required hospitalization, with a mean length of stay of 5 days (range: 3–10 days), and no cases required intensive care. One patient (0.2%) underwent surgery for a paranasal abscess. There were no significant differences in hospitalization rates between the different age groups (χ2 = 1.30, *p* = 0.861) or genders (χ2 = 0.09, *p* = 0.766). Pneumonia, along with other respiratory tract diseases, was the most common condition requiring inpatient admission. Importantly, high levels of satisfaction were reported with both the referral process and the medical care received at external medical centers.

Previous studies, primarily in adult populations, have explored similar approaches to referring low-acuity patients [18–21]. Van der Straten et al. demonstrated that such patients could be safely managed in doctor’s offices after referral from acute care posts, reporting a hospitalization rate of 1.2%, closely aligning with our findings. Notably, nearly 25% of patients triaged as more urgent (UL-3–4) were also referred to a doctor’s office, with a hospitalization rate of 4.0% [12]. Morreel et al. further examined referral safety and reported a slightly higher hospitalization rate (2.4%), compared to our findings [22].

Another study highlighted challenges with caregivers’ compliance, with nearly one-fifth refusing referrals [13], unlike in our study, where clear communication strategies mitigated this issue. Within our study population, 478 patients (84.1%) followed the recommendation received at our PED to visit either a pediatric office or a hospital close to their home. This high adherence rate could reflect the clear instructions given by the triage nurses and the general compliance of the families to follow the recommendations for medical visits at primary and secondary care centers. Nevertheless, 56 patients (9.9%) did not proceed with the recommendation, which should be avoided due to the residual risk of delayed or missed diagnosis. Therefore, it is essential to provide clear information about the MTS, the reason for the referral, and the importance of attending a recommended medical visit.

Previously, other studies have explored approaches to alleviate overall burden on EDs, while maintaining high patient satisfaction levels [23–25]. In terms of perception, parents/caregivers of 530 patients (93.3%) expressed satisfaction with the MTS assessment and procedure in our PED, and the guardians of 477 patients (84.0%) were satisfied with the treatment provided in the external medical care centers. These findings indicate a generally positive perception of the triage-referral strategy. Notably, a slightly higher number of families reported dissatisfaction with the procedure in our PED compared to external medical centers (*n* = 26, 4.6% vs. *n* = 22, 3.9%). The dissatisfaction with the PED originated mostly from insufficient information provided about the triage procedure and the fact of being referred.

While patient safety is the primary focus, the effectiveness of a triage-referral system also relies on parents’/caregivers’ cooperation and understanding. Clear communication and education about triage protocols are essential to ensure adherence, reduce misuse of emergency services, and help to prevent aggression toward medical staff. Importantly, the MTS is intended for use in health care centers, where trained staff are available.

These findings contribute to the ongoing worldwide discussion about mitigating ED overcrowding to streamline care delivery and thereby minimize potential harm to patients [26, 27]. Possible solutions include implementation of international standardized triage systems that prioritize the urgency of each case based on the severity of the patients’ conditions, integrating health information technologies [28] to streamline processes and enhancing coordination between EDs and other healthcare services to facilitate patient referral [8, 18, 29–33]. Improving staff training, public education campaigns [29, 30, 34], and higher investments in pediatric primary care [35] were also described to be critical in the efficient management of patient overcrowding in EDs.

## Conclusion

We conclude that the MTS provides a valuable tool to reduce overcrowding in PEDs, especially of a tertiary care center by referring low-triaged patients to local pediatric offices and hospitals for physical examination and treatment. This approach emphasizes the importance of clearly informing patients and their parents/caregivers about the urgency level, doctors’ decision, and clear referral instructions.

To enhance patient safety, the referrals are not allowed for newborns, high-risk patients with chronic illnesses, those with infectious diseases, and also for all cases if the maximum waiting time for the doctors’ decision on referral exceeds 15 min and involves major language barriers. It is important that the decision for referral is made by pediatric specialists with experience in managing complex pediatric cases with and without critical illnesses.

Our data show that most patients and their parents/caregivers follow the instructions of the triage nurses and go to an external medical center. Although one-quarter expressed dissatisfaction with the provided information, patient safety seemed to be maintained. These findings support the implementation of the triage-referral strategy in tertiary care centers by referring low-urgency patients for physical examinations and treatment and at the same time contribute to the effectiveness of PEDs for critically ill patients.

### Limitations and perspectives

Although referral decisions were based on MTS triage data and predefined exclusion criteria, the final decision was made by the responsible PED physician and may carry a degree of subjectivity. Detailed data on the clinical reasons for non-referral decisions by PED physicians were not prospectively recorded, which limits insight into the full scope of the decision-making process. Another limitation of this study is the relatively small number of patients who participated in the survey. A substantial proportion of patients were lost to follow-up (76.3%), which may limit the generalizability of our findings. However, comparative analysis of demographic and clinical characteristics between the study population and those lost to follow-up did not reveal statistically significant differences, suggesting that the included cohort is broadly representative of the overall referred population. The responses and diagnoses were only provided by parents/caregivers, but not by healthcare professionals, which could be a potential source of bias and false/inaccurate information. Telephone call surveys were performed by medical students, whose experience in clinical studies and pediatric illnesses may have been limited. Although language barriers were minimized by the medical students speaking different native languages, not all languages could be accommodated.

In summary, the findings of this study highlight the potential of structured triage-based referral pathways to safely manage low-urgency pediatric patients in tertiary PED settings. Importantly, the results support the feasibility of implementing such referral systems without compromising patient safety, when decisions are made by experienced pediatric physicians based on triage documentation. Given the observational nature of this study and the relatively low number of adverse outcomes, future research should focus on prospective validation of triage-based referral protocols. Further studies may also explore long-term outcomes, cost-effectiveness, and the impact on healthcare resource utilization.

## Data Availability

The anonymised data supporting this study’s findings are available upon reasonable request to the corresponding author, SGP. The data are not publicly available due to privacy and ethical restrictions.

## Abbreviations

ED: Emergency department
ICU: Intensive care unit
MTS: Manchester Triage System
PED: Pediatric emergency department
UL: Urgency level
